# Creation of a recombinant Komagataella phaffii strain, a producer of proteinase K from Tritirachium album

**DOI:** 10.18699/VJ21.102

**Published:** 2021-12

**Authors:** A.B. Beklemishev, M.B. Pykhtina, Ya.M. Kulikov, T.N. Goryachkovskaya, D.V. Bochkov, S.V. Sergeeva, A.R. Vasileva, V.P. Romanov, D.S. Novikova, S.E. Peltek

**Affiliations:** Federal Research Center of Fundamental and Translational Medicine, Novosibirsk, Russia; Institute of Cytology and Genetics of the Siberian Branch of the Russian Academy of Sciences, Novosibirsk, Russia 3 Efirnoe; Federal Research Center of Fundamental and Translational Medicine, Novosibirsk, Russia; Institute of Cytology and Genetics of the Siberian Branch of the Russian Academy of Sciences, Novosibirsk, Russia 3 Efirnoe; Federal Research Center of Fundamental and Translational Medicine, Novosibirsk, Russia; Institute of Cytology and Genetics of the Siberian Branch of the Russian Academy of Sciences, Novosibirsk, Russia 3 Efirnoe; Institute of Cytology and Genetics of the Siberian Branch of the Russian Academy of Sciences, Novosibirsk, Russia; Institute of Cytology and Genetics of the Siberian Branch of the Russian Academy of Sciences, Novosibirsk, Russia; Institute of Cytology and Genetics of the Siberian Branch of the Russian Academy of Sciences, Novosibirsk, Russia; Institute of Cytology and Genetics of the Siberian Branch of the Russian Academy of Sciences, Novosibirsk, Russia; Institute of Cytology and Genetics of the Siberian Branch of the Russian Academy of Sciences, Novosibirsk, Russia; Efirnoe Joint-Stock Company, Alekseyevka, Belgorod region, Russia; Institute of Cytology and Genetics of the Siberian Branch of the Russian Academy of Sciences, Novosibirsk, Russia

**Keywords:** proteinase K, gene cloning, Komagataella phaffii, gene expression, enzymatic activity, протеиназа К, клонирование гена, Komagataella phaffii, экспрессия гена, активность фермента

## Abstract

The objects of the study were recombinant clones of Komagataella phaffii K51 carrying the heterologous proteinase K (PK-w) gene from Tritirachium album integrated into their genome as well as samples of recombinant proteinase K isolated from these clones. The aims of this work were i) to determine whether it is possible to create recombinant K. phaffii K51 clones overexpressing functionally active proteinase K from T. album and ii) to analyze the enzymatic activity of the resulting recombinant enzyme. The following methods were used: computational analysis of primary structure of the proteinase K gene, molecular biological methods (PCR, electrophoresis of DNA in an agarose gel, electrophoresis of proteins in an SDS polyacrylamide gel under denaturing conditions, spectrophotometry, and quantitative assays of protease activity), and genetic engineering techniques (cloning and selection of genes in bacterial cells Escherichia coli TOP10 and in the methylotrophic yeast K. phaffii K51). The gene encoding natural proteinase K (PK-w) was designed and optimized for expression in K. phaffii K51. The proteinase K gene was synthesized and cloned within the plasmid pPICZα-A vector in E. coli TOP10 cells. The proteinase K gene was inserted into pPICZα-A in such a way that – at a subsequent stage of transfection into yeast cells – it was efficiently expressed under the control of the promoter and terminator of the AOX1 gene, and the product of the exogenous gene contained the signal peptide of the Saccharomyces cerevisiae a-factor to ensure the protein’s secretion into the culture medium. The resultant recombinant plasmid (pPICZα-A/PK-w) was transfected into K. phaffii K51 cells. A recombinant K. phaffii K51 clone was obtained that carried the synthetic proteinase K gene and ensured its effective expression and secretion into the culture medium. An approximate productivity of the yeast recombinant clones for recombinant proteinase K was 25 μg/ mL after 4 days of cultivation. The resulting recombinant protease has a high specific proteolytic activity: ~5000 U/mg.

## Abstract (ru)

Объектами исследования являлись рекомбинантные штаммы Komagataella phaffii K51, несущие интегрированный в их геном гетерологичный ген протеиназы К (PK-w) из Tritirachium album, а также препарат рекомбинантной протеиназы К, полученный из этих штаммов. Целью работы было изучение возможности по- лучения рекомбинантных штаммов K. phaffii K51, обеспечивающих высокий уровень синтеза функционально активной протеиназы К из T. album, и анализ ферментативной активности полученного рекомбинантного эн- зима. В работе использованы методы компьютерного анализа первичной структуры гена протеиназы К, моле- кулярно-биологические методы (ПЦР, электрофорез ДНК в агарозных гелях, электрофорез белков в SDS-ПААГ в денатурирующих условиях, спектрофотометрия, методы количественного определения активности протеаз), генно-инженерные методы (методы клонирования и селекции генов в бактериальных клетках Escherichia coli str. TOP10 и в метилотрофных дрожжах K. phaffii str. K51). Спроектирован ген природной протеиназы К (PK- w), оптимизированный для экспрессии в дрожжах K. phaffii K51. Осуществлены синтез и клонирование синтезиро- ванного гена протеиназы К в составе вектора pPICZα-A в клетках E. coli str. TOP10. Ген протеиназы К встроен в векторную плазмиду pPICZα-A таким образом, чтобы на последующем этапе переклонирования в клетках дрожжей обеспечить его эффективную экспрессию под контролем промотора и терминатора гена AOX1, а про- дукт экспрессии клонированного гена содержал сигнальный пептид альфа-фактора Saccharomyces cerevisiae для обеспечения секреции белка в культуральную жидкость. Проведено переклонирование рекомбинантной плазмиды (pPICZα-A/PK-w) в клетках дрожжей K. phaffii str. K51. Получен рекомбинантный штамм K. phaffii K51, несущий синтетический ген протеиназы К и обеспечивающий его экспрессию в дрожжах и секрецию в культу- ральную среду. Приблизительный выход рекомбинантной протеиназы К после четырех суток культивирования дрожжевых рекомбинантных клонов составил 25 мкг/мл. Полученный препарат рекомбинантной протеазы об- ладает высокой удельной протеолитической активностью, составляющей ~5000 Ед/мг.

## Introduction

More than 70 % of enzymes used in various fields of industry are hydrolases (Kudryavtseva et al., 2008; Yin et al., 2014), and proteases account for more than 30 % of the total market of industrial enzymes (Kulkarni et al., 1999; Gupta et al., 2002; Koga et al., 2014; Singh et al., 2016). This is due to their widespread use in various areas of industry, in particular in the production of detergents, in waste disposal, and in the food, dairy, leather, pharmaceutical, and textile industries. The increased demand for the production of proteases in recent years is caused by the urgent need to manufacture high-quality effective detergents as well as new food products from agricultural waste of plant raw materials and from waste of meat and fish processing. In the past three decades, proteinases from various sources (bacteria, bacilli and fungi) have found many applications in various fields of industry and in clinical practice. The most studied group of proteolytic enzymes is bacterial serine proteinases. They are actively used in the pharmaceutical industry, in tissue engineering, and systemic enzyme therapies (Gupta et al., 2002; Kudryavtseva et al., 2008; Yin et al., 2014).

Formulations containing proteinases are widely employed in many fields of medicine: in surgery – for the treatment of trophic ulcers, abscesses, phlegmons, osteomyelitis, and other purulent-inflammatory processes; in dentistry – for the treatment of caries, pulpitis, periodontitis, periodontal disease, and its complications, and in pulmonology – as a mucolytic drug for the treatment of various types of pneumonia and bronchitis (administration via inhalation).

Numerous independent studies confirm that serine proteases hold promise for medical purposes (Yariswamy et al., 2013; Muthu et al., 2017; Belov et al., 2018; Abaturov, 2020; Osmolovsky et al., 2020). In particular, one of the main problems that physicians face when treating skin wounds and burns in people with compromised immunity is the formation of a surface biofilm generated by conditionally pathogenic microorganisms (Staphylococcus aureus and S. epidermidis, micrococci, and Pseudomonas); under this biofilm, the microbes cannot be reached by antibiotics, and as a consequence, wound healing slows down.

For the degradation of various components of the biofilm extracellular matrix, various formulations are currently being designed based on a mixture of enzymes: proteases (including proteinase K), glycosidases, and deoxyribonucleases (Abaturov, 2020). One of the promising areas for application of proteinases is the creation of thrombolytic drugs on the basis of these enzymes. Thus, the development of new effective therapeutics based on enzymes of bacterial origin has good potential for modern medicine, microbiology, and biotechnology.

Thermostable proteases are the most popular in this regard because, firstly, they are characterized by a higher rate of catalysis, and secondly, they provide protection of the reaction mixture and products of enzymatic conversion from microbial contamination because these enzymes catalyze the reactions at high temperatures. Both bacterial and yeast strains that are recombinant superproducers of thermostable proteases have been constructed, and in most studies, it has been shown that the methylotrophic yeast Komagataella phaffii generates a larger amount of recombinant proteases than bacterial strains do (Kim et al., 2005; Latiffi et al., 2013; Yu et al., 2014; Ma et al., 2016; Shu et al., 2016; Kangwa et al., 2018; Pereira et al., 2020). In addition, the proteases produced by yeasts are usually secreted into the culture medium in a soluble functionally active state (Yang et al., 2016). Of particular interest are proteinases that exert their activity in a wide range of temperatures and pH of the medium.

Accordingly, a solution to the problem of obtaining a yeast superproducer of a proteinase from Tritirachium album (proteinase K) is undoubtedly intriguing because this proteinase has a number of important practical advantages: it has broad specificity, is most active at high reaction temperatures (37 to 60 °C), is functional across a wide pH range (4–12), and is not inhibited by ionic or nonionic detergents. The present study is aimed at solving this problem

## Materials and methods

Materials.

All chemical reagents of analytical purity were purchased from Sigma-Aldrich (USA) or Reachem (Moscow, Russia), and restriction endonucleases from SibEnzyme (Novosibirsk, Russia). DNA ligase T4 and DNA polymerase Phusion were acquired from Thermo Fisher Scientific Inc. (USA), and oligonucleotides – Biosintez (Novosibirsk, Russia). Yeast extract, bactopeptone, and tryptone from Difco were utilized to prepare the Luria-Bertani (LB) medium for growing Escherichia coli cells. Yeast culture media (YPD, BMGY, BMM2, and BMM10) were prepared as described in the manufacturer’s protocol (Easy Select™ Pichia Expression Kit (Invitrogen, USA). Modified Eagle medium (MEM) was bought from Biolot (Russia), dithiothreitol and iodoacetamide, from Bio-Rad (USA), and porcine trypsin, from Promega (Trypsin Gold, Mass Spectrometry Grade, USA). DEAESepharose FF and SP Sepharose FF ion exchange resins were purchased from GE Healthcare Bioscience (Sweden). The water used in the work was deionized and autoclaved.

Bacterial and yeast strains and plasmid vectors. The yeast K. phaffii K51 strain was obtained from the Russian National Collection of Industrial Microorganisms (cat. No. Y-4935), whereas E. coli TOP10 and the pPICZα-A plasmid vector were acquired from Invitrogen Inc. (USA).

Buffers and culture media. Solutions and buffers were prepared from deionized autoclaved water. E. coli clones carrying the pPICZα-A plasmid or its derivatives were selected on low-salt LB agar plates (1 % of tryptone, 0.5 % of yeast extract, 0.5 % of NaCl, 1.8 % of Bacto-agar, and 50 μg/mL zeocin). Yeast cells were grown in the YPD medium (2 % of yeast peptone, 1 % of yeast extract, and 2 % of dextrose). Yeast transformants were cultured and selected on YPD agar plates (2 %) with various concentrations of zeocin (500 or 2000 μg/ mL). The selected yeast clones were also cultivated in the BMGY medium (1 % of yeast extract, 2 % of peptone, 100 mM potassium phosphate pH 6.0, 1.34 % of YNB, 4 × 10–5 % of biotin, and 2 % of glycerol). To induce the AOX1 gene promoter, the clones were cultivated first in the BMM2 medium (1.34 % of YNB, 4 × 10–5 % of biotin, and 1 % of methanol) and then in the BMM10 medium (1.34 % of YNB, 4 × 10–5 % of biotin, and 5 % of methanol).

Construction of recombinant plasmid (pPICZα-A/ PK- w). The nucleotide sequence of a synthetic gene encoding natural proteinase K from T. album (i. e., protease K, endopeptidase K; E.C.3.4.21.64) – hereinafter referred to as PK-w – was designed and optimized for expression in the yeast K. phaffii. The optimized PK-w protease gene was synthesized by GenScript (USA). The PK-w protease gene was cloned in E. coli cells after insertion into the pPICZα-A plasmid at the XhoI and XbaI sites.

Small-scale preparation of recombinant proteinase K (PK-w). The genetically modified yeast strain was grown in 250 mL of the BMGY medium with 1 % of glycerol in 1 L flasks on an orbital shaker at 250 rpm for 48 h at 28 °C. Next, protein biosynthesis was induced with 1 % methanol (every day, 25 mL of 10 % methanol was added) for 4 days. On the 4th day, proteolytic activity in the culture liquid was determined.

The protein concentration in solutions was determined by three methods: a) via absorption measurement in the protein solution at 280 nm, taking into account the extinction coefficient for the protein; b) by densitometry of the colored protein band in a gel; c) by the Bradford assay with the Quick Start™ Bradford 1×Dye Reagent (Bio-Rad) according to the manufacturer’s instructions.

Determination of protease activity of the recombinant proteinase K toward casein. Proteolytic activity was determined by the Kunitz method using casein from cow’s milk (Sigma-Aldrich) as a substrate (Bisswanger, 2010). For this purpose, 0.4 mL of a 2 mg/mL casein solution in 10 mM Tris- HCl buffer (pH 8.0) containing 10 mM CaCl2 was heated to 55 °С, and 0.2 mL of the enzyme solution in the same buffer was added. The mixture was incubated at 55 °С for 10 min, and then the reaction was stopped by the addition of 1 mL of 1.2 M trichloroacetic acid. A control solution was subjected to the same procedures, except that the enzyme solution was introduced into the casein solution after the addition of trichloroacetic acid. The samples were centrifuged at 10 000 g for 5 min at 5 °С, and supernatant absorbance was determined at a wavelength of 275 nm. One unit of activity was defined as the amount of the protease that leads – in 1 min at 55 °С – to the same absorption value as does 1 μmol of tyrosine (according to a calibration curve). The calibration curve was built within the appropriate range of tyrosine concentrations.

Quantitation of protease activity in the culture liquid toward azocasein. A culture liquid was centrifuged at 4 °C for 10 min at 10 000 g to pellet the cells, and the supernatant was collected for the protease activity assay.

The reaction mixture consisting of 0.5 mL of a 0.2 % solution of azocasein in 50 mM Tris-glycine buffer (pH 8) and 0.25 mL of the supernatant was heated in a water bath at 55 °C for 40 min. The reaction was stopped by the addition of 1 mL of 1.2 M trichloroacetic acid. A control solution containing 0.5 mL of a 0.2 % solution of azocasein in 50 mM Tris-glycine buffer (pH 8) without the supernatant was heated in a water bath at 55 °C for 40 min. The reaction was stopped by the addition of 1 mL of 1.2 M trichloroacetic acid, after which 0.25 mL of the supernatant was introduced into the mixture. The product of the azocasein hydrolysis was quantified spectrophotometrically by means of absorption at 440 nm.

## Results

Design of the plasmid for the expression of the proteinase K gene in the yeast K. phaffii K51

**Fig. 1.**
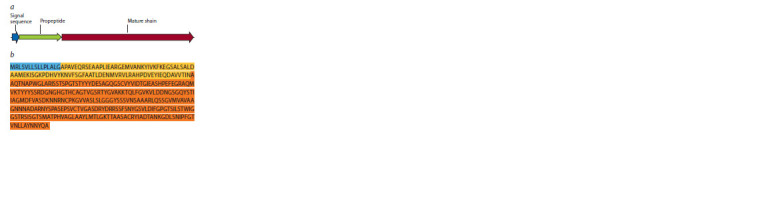
Domain structure of proteinase K (a) and amino acid sequence of the PK-w precursor protein (b). The signal peptide is highlighted in blue, the prepeptide (prodomain) is highlighted in yellow, and the mature protein is orange.

To obtain a yeast producer of proteinase K (i. e., protease K, endopeptidase K; E.C. 3.4.21.64), the proteinase gene from T. album was optimized for expression in the methylotrophic yeast K. phaffii K51. The amino acid sequence of the proteinase K precursor protein and the layout of its domains are shown in Fig. 1.

The synthesized gene encoded only the prepeptide and amino acid sequence of the mature protein. The signal peptide of the Saccharomyces cerevisiae α-factor encoded by a fragment of the pPICZα-A vector (data not shown) served as a signal for enzyme secretion.

Cloning of the recombinant plasmid pPICZα-A/PK-w in E. coli TOP10 cells

Electrocompetent E. coli TOP10 cells were transformed with recombinant plasmids (pPICZα-A/PK-w) using an electroporator (Bio-Rad). The transformed cells were inoculated into 1 mL of the LB medium and incubated at 37 °C for 1 h on an orbital shaker at 140 rpm. The cell suspensions were plated on agar Petri dishes containing 50 μg/mL zeocin and incubated for 16 h at 37 °C. From 150 to 200 colonies emerged in each plate. Ten colonies from each plate were transferred (with puncturing) to separate Petri dishes containing the agar low-salt LB medium with 50 μg/mL zeocin and were utilized to prepare thermolysates for colony PCR detecting the recombinant plasmid (pPICZα-A/PK-w). The PCR was carried out with a pair of primers specific for the regions of the pPICZα-A vector flanking the inserted gene: forward primer No. 324-AOX1-F, 5′-GACTGGTTCCAATTGACAAGC-3′; and reverse primer No. 325-AOX1-R, 5′-GCAAATGGCATTCTGACATCC-3′. Amplicon sizes were analyzed by electrophoresis in a 0.8 % agarose gel containing ethidium bromide. PCR-positive clones were selected for subsequent lab scale production of recombinant plasmids with the aim of their subsequent transfection into yeast cells

**Fig. 2.**
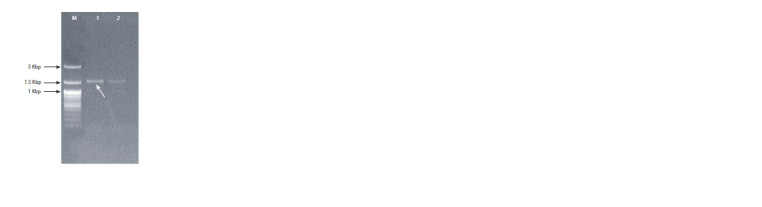
Electropherogram of the PCR products obtained via amplification of a portion of the vector containing the proteinase K gene (designated as the pPICZα-A/PK-w plasmid). The arrow indicates the DNA fragment used for subsequent work. Lanes: M – SibEnzyme DNA molecular weight markers (100–3000 bp); 1 and 2 – proteinase K gene amplicons from two recombinant clones carrying plasmids with the PK-w gene insert.

Fig. 2 shows the results of testing E. coli TOP10 clones by colony PCR for the presence of the pPICZα-A plasmid carrying an insert of the proteinase K gene (named as the pPICZα-A/PK-w plasmid).

As readers can see in Fig. 2, the amplicons obtained from two recombinant plasmids containing the inserted proteinase K gene have the theoretically expected size: ~1626 bp. The selected clone containing the pPICZα-A/PK-w plasmid with the insertion of the proteinase K gene (PK-w) was used for further procedures: lab scale production of the plasmid, its linearization with restriction endonuclease BstX1, and subsequent transfection into K. phaffii K51 yeast cells.

Transfection of the proteinase K gene into K. phaffii K-51 yeast cells and screening of transfectants

At the first stage, the selected E. coli clone was propagated in 100 mL of the LB medium, followed by isolation of pPICZα-A/PK-w plasmid DNA from the cells by means of the GenElute™ HP Plasmid Midiprep Kit. The isolated plasmid DNA was analyzed by electrophoresis in a 0.8 % agarose gel stained with ethidium bromide.

a Qubit fluorometer (Invitrogen). As a result of the isolation procedure, ~25 μg of the purified pPICZα-A/PK-w plasmid was obtained. Approximately 5–10 μg of the isolated pPICZα-A/PK-w plasmid was linearized by digestion with restriction endonuclease BstX1 and used for transfection (electroporation of K. phaffii K51, see below). At the end of the restriction reaction, phenol-chloroform extraction of DNA was carried out, followed by its precipitation with isopropanol and washing with 70 % ethanol. DNA pellets were dissolved in 10 μL of double-distilled H2O, frozen, and stored at –20 °C. The completeness of the plasmid hydrolysis reaction was verified by electrophoresis of restriction products in a 0.8 % agarose gel stained with ethidium bromide. Judging by the results of the electrophoretic analysis, the bulk of the plasmid sample was successfully hydrolyzed by restriction endonuclease BstXI. For the transformation of electrocompetent K. phaffii K51 cells, 13–15 μg of plasmid DNA was employed, dissolved in 10 μL of double-distilled H2O. Electroporation was performed using a Gene Pulser Xcell Total System Electroporator (Bio-Rad).

After preliminary cultivation on an orbital shaker at 200 rpm for 2 h at 27 °C in test tubes containing 1 mL of the YPD medium, the transformed cells were plated on Petri dishes with the agar YPD medium containing 500 or 2000 μg/mL zeocin. The culture plates were placed in a thermostat at 30 °C for 3–5-day incubation. On the 4th day after the cell transfection with the pPICZα-A/PK-w plasmid, many separate colonies were visible on the culture plates with 500 μg/mL of zeocin, whereas on the plates with 2000 μg/mL zeocin, there were 50 to 100 colonies. Such a large number of colonies on the plates with 2000 μg/mL zeocin is apparently due to partial degradation of the antibiotic, which had been stored at 4 °C for a long time.

Zeocin-resistant transformants grown on the culture plates with 2000 μg/mL zeocin were evaluated for their ability to synthesize and secrete the desired protein: we cultured the selected clones in 96-well deep well plates (Axygen Scientific). For screening, 20 colonies were randomly chosen, which were placed into the wells of these plates. In parallel, the same colonies, grown on agar plates with a zeocin concentration of 2000 μg/mL, were transferred to separate Petri dishes (containing an agar medium with the same zeocin concentration) by puncturing sites labeled with numbers.

The chosen clones were cultivated individually in 96-well deep well plates in 300 μL of the BMGY medium on an orbital shaker at 250 rpm for 48 h at 28 °C. Then, 250 μL of the BMM2 medium was added into each well. On each of the next 3 days, 50 μL of the BMM10 medium was introduced into the wells. On day 4, the culture liquid from each well was centrifuged at 6000 rpm for 5 min to pellet the cells, and the resulting supernatants were analyzed by SDS-PAGE for the presence of the target protein.

**Fig. 3.**
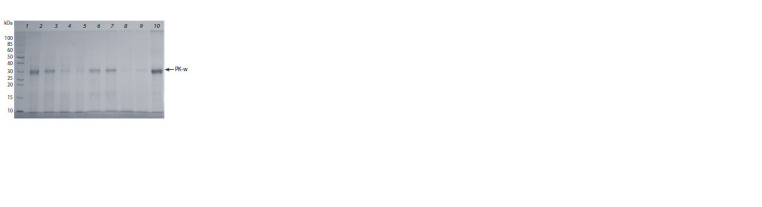
An electropherogram of the proteins produced by the tested yeast clones transformed with the pPICZα-A/PK-w plasmid. Lanes: 1 – Thermo Scientific Molecular Weight Markers (10–200 kDa); 2–10 – proteins produced and secreted by the analyzed clones.

Samples for the electrophoresis were prepared as follows: 10 trichloroacetic acid was added to the supernatants to concentrate the proteins. The protein precipitates were washed with acetone, resuspended in 1× TGB, and 4× denaturing buffer was added, followed by boiling of the samples, and then the proteins were separated in a 12.5 % polyacrylamide gel. According to the electrophoresis results, we chose culture liquids of the clones producing the largest amount of proteins with a molecular weight corresponding to that of natural proteinase K (~30 kDa). The purpose was to determine the concentration of recombinant proteinase K and assess its enzymatic activity. The results of electrophoretic analysis of proteins produced and secreted by the recombinant yeast clones are depicted in Fig. 3.

**Fig. 4.**
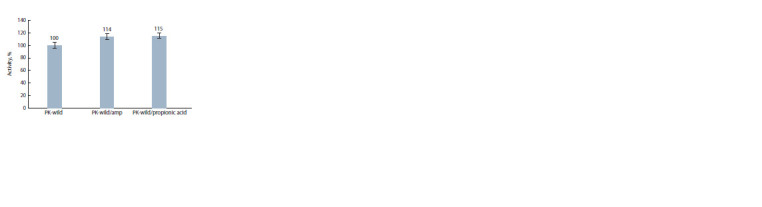
Proteolytic activity of the culture liquid of recombinant yeast clone No. 10 as a result of its small-scale propagation in the presence of ampicillin (PK-wild/amp) or propionic acid (PK-wild/propionic acid) or without any antibacterial agents (PK-wild). The proteolytic activity of the culture liquid obtained without the antibacterial agents (PK-wild) was set to 100 %.

As presented in Fig. 4, all zeocin-selected clones produce a major protein with a molecular weight of ~29–30 kDa, which corresponds to the molecular weight of mature proteinase K: 28 903 Da. According to the electrophoresis results, clone No. 10 was chosen for further experiments because it produced the largest amount of a recombinant protein with a molecular weight of ~29–30 kDa. The yield of recombinant protease PK-w after 4 days of cultivation of recombinant yeast clone No. 10 in the 96-well plate was 25 μg/mL.

Propionic acid was applied to control bacterial contamination during lab scale production of large amounts of the recombinant protein. Fig. 4 shows that the use of propionic acid at a concentration of 0.025 % is comparable to the addition of ampicillin at 0.2 mg/mL.

Microscopic analysis of the cultures did not reveal the presence of bacteria; however, the use of ampicillin and propionic acid somewhat increased the production of the target protein.

Recombinant yeast clone No. 10 was utilized for preliminary small-scale preparation of the enzyme.

Quantification of the protease activity of the recombinant protein produced by clone No. 10

As a result of these measurements, it was found that the obtained batch of the recombinant protein with a molecular weight of ~29–30 kDa had a high specific proteolytic activity, ~5000 U/mg. This finding indicated that this recombinant protein is proteinase K (PK-w).

**Fig. 5.**
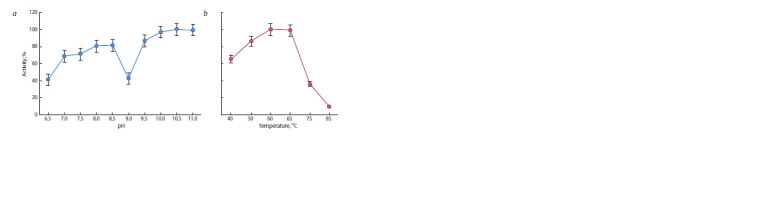
Dependence of the recombinant proteinase K’s activity on pH and temperature.

The dependence of the obtained recombinant proteinase K’s activity on pH of the medium and on reaction temperature was investigated next (Fig. 5). The optimum of enzymatic activity of the obtained recombinant proteinase K is in the range of pH 10–11, although the enzyme is also active at pH from 9.5 to 6.5 (see Fig. 5, a). A sharp drop of the activity was observed at pH 9.0 and at pH ≤ 4.0. The optimal temperature range for the manifestation of this protease activity turned out to be 40–65 °C (see Fig. 5, b).

Chromatographic purification of the obtained recombinant proteinase K

All procedures were conducted at a temperature ≤ 5 °C. The culture fluid of recombinant clone No. 10 was separated from the cells by centrifugation for 25 min at 4000 rpm. Lowmolecular- weight impurities were removed and the supernatant was concentrated 20-fold via ultrafiltration by means of centrifugal concentrators.

Protein impurities were removed from the enzyme sample by ion exchange chromatography on anion exchange resin DEAE-Sepharose 6HF. Elution was performed with a buffer composed of 50 mM sodium chloride and 50 mM Tris- HCl (pH 7.2). Fractions showing proteolytic activity were pooled, concentrated using the centrifuge concentrators, and either lyophilized or stored in 50 % glycerin in a freezer of a fridge.

**Fig. 6.**
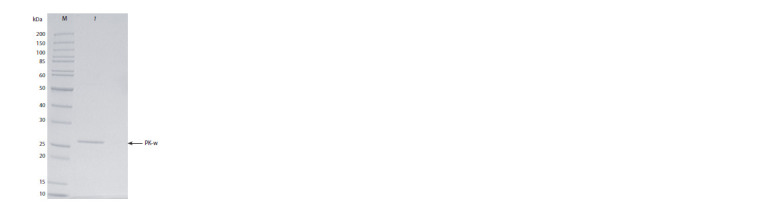
An electropherogram of the purified recombinant protein from the batch produced by yeast clone No. 10. Electrophoresis was performed in an SDS 12.5 % polyacrylamide gel. Lanes: M – Thermo Scientific Molecular Weight Markers (10–200 kDa); 1 – the chromatographically purified recombinant protein.

The purified recombinant protein was analyzed by electrophoresis in SDS 12.5 % polyacrylamide gel (Fig. 6). As presented in Fig. 6, on the gel, the recombinant protein is represented by one major band with a size in the range of ~26.5–27.0 kDa. There are no protein impurities on the gel. The activity of the purified protein was 49 800 U/mg toward azocasein and 5000 U/mg toward casein. Thus, a highly purified batch of recombinant proteinase K was obtained.

## Conclusion

The design and optimization of the nucleotide sequence encoding the precursor protein of natural proteinase K (PK-w) from T. album were performed to ensure its efficient expression in the yeast K. phaffii. The synthesized proteinase K gene was cloned within the pPICZα-A vector in E. coli str. TOP10 cells, and then the plasmid was isolated and transfected into yeast K. phaffii str. K51 cells.

A recombinant clone of K. phaffii K51 that carries the gene of recombinant proteinase K and successfully expresses and secretes this enzyme into the culture medium was obtained. A lab scale batch of the recombinant proteinase K was prepared. Protease activity of the obtained recombinant proteinase K (PK-w) was determined with casein and azocasein as substrates. The enzyme batch has a high specific proteolytic activity: ~5000 U/mg. The optimal enzymatic activity of the obtained recombinant proteinase K is in a pH range of 10–11 and a temperature range of 40–65 °C.

## Conflict of interest

The authors declare no conflict of interest.
